# Diaqua­(5,10,15,20-tetra­phenyl­porphyrinato-κ^4^ *N*)magnesium–18-crown-6 (1/1)

**DOI:** 10.1107/S1600536813001219

**Published:** 2013-01-19

**Authors:** Khaireddine Ezzayani, Soumaya Nasri, Mohamed Salah Belkhiria, Jean-Claude Daran, Habib Nasri

**Affiliations:** a Laboratoire de Physico-chimie des Matériaux, Université de Monastir, Faculté des Sciences de Monastir, Avenue de l’environnement, 5019 Monastir, Tunisia; b Laboratoire de Chimie de Coordination, CNRS UPR 8241, 205 route de Narbonne, 31077 Toulouse, Cedex 04, France

## Abstract

In the title compound, [Mg(C_44_H_28_N_4_)(H_2_O)_2_]·C_12_H_24_O_6_, the Mg^II^ cation lies on an inversion center and is octa­hedrally coordinated by the four N atoms of the deprotonated tetra­phenyl­porphyrin (TPP) ligand and by two water mol­ecules. The asymmetric unit contains one half of the [Mg(TPP)(H_2_O)_2_] complex and one half of an 18-crown-6 mol­ecule. The average equatorial magnesium–pyrrole N atom distance (Mg—N_p_) is 2.071 (1) Å and the axial Mg—O(H_2_O) bond length is 2.213 (1) Å. The crystal packing is stabilized by two O—H⋯O hydrogen bonds between coordinating water mol­ecules and adjacent 18-crown-6 mol­ecules, and exhibits a one-dimensional supramolecular structure along the *a* axis. The supramolecular architecture is futher stabilized by weak C—H⋯π inter­actions. The 18-crown-6 mol­ecule is disordered over two sets of sites with an occupancy ratio of 0.8:0.2.

## Related literature

For general background to magnesium porphyrin species and their applications, see: Ghosh *et al.* (2010 ▶). For related structures, see**:** Belghith *et al.* (2012 ▶); McArdle (1995 ▶); McKee *et al.* (1984 ▶); Choon *et al.* (1986 ▶); McKee & Rodley (1988 ▶); Gryz *et al.* (2007 ▶); Imaz *et al.* (2005 ▶). For a description of the Cambridge Structural Database, see: Allen (2002 ▶). 
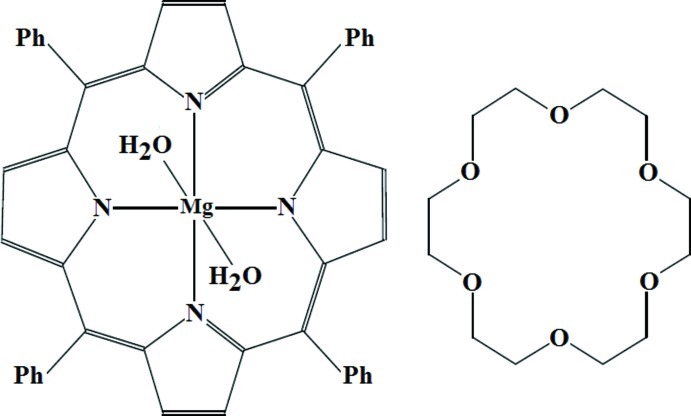


## Experimental

### 

#### Crystal data

[Mg(C_44_H_28_N_4_)(H_2_O)_2_]·C_12_H_24_O_6_
*M* *_r_* = 937.36
Triclinic, 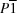

*a* = 8.1440 (3) Å
*b* = 12.3080 (4) Å
*c* = 12.4170 (4) Å
α = 86.894 (3)°
β = 75.163 (3)°
γ = 79.529 (3)°
*V* = 1183.06 (7) Å^3^
*Z* = 1
Mo *K*α radiation
μ = 0.10 mm^−1^
*T* = 180 K
0.56 × 0.51 × 0.19 mm

#### Data collection

Oxford Diffraction Xcalibur (Sapphire1) diffractometer
Absorption correction: multi-scan (*CrysAlis RED*; Oxford Diffraction, 2009 ▶) *T*_min_ = 0.946, *T*_max_ = 0.981
23613 measured reflections
4650 independent reflections
4013 reflections with *I* > 2σ(*I*)
*R*_int_ = 0.031

#### Refinement

*R*[*F* ^2^ > 2σ(*F* ^2^)] = 0.044
*wR*(*F* ^2^) = 0.118
*S* = 1.04
4650 reflections
400 parameters
119 restraints
H atoms treated by a mixture of independent and constrained refinement
Δρ_max_ = 0.46 e Å^−3^
Δρ_min_ = −0.28 e Å^−3^

### 

Data collection: *CrysAlis CCD* (Oxford Diffraction, 2009 ▶); cell refinement: *CrysAlis RED* (Oxford Diffraction, 2009 ▶); data reduction: *CrysAlis RED*; program(s) used to solve structure: *SIR2004* (Burla *et al.*, 2005 ▶); program(s) used to refine structure: *SHELXL97* (Sheldrick, 2008 ▶); molecular graphics: *ORTEPIII* (Burnett & Johnson, 1996 ▶) and *ORTEP-3 for Windows* (Farrugia, 2012 ▶); software used to prepare material for publication: *SHELXL97*.

## Supplementary Material

Crystal structure: contains datablock(s) I, global. DOI: 10.1107/S1600536813001219/xu5669sup1.cif

Structure factors: contains datablock(s) I. DOI: 10.1107/S1600536813001219/xu5669Isup2.hkl

Additional supplementary materials:  crystallographic information; 3D view; checkCIF report

## Figures and Tables

**Table 1:** Hydrogen-bond geometry (Å, °) *Cg*2 and *Cg*4 are the centroids of the N2/C6–C9 and C17–C22 rings, respectively.

*D*—H⋯*A*	*D*—H	H⋯*A*	*D*⋯*A*	*D*—H⋯*A*
O1—H1*O*1⋯O2*A*	0.97 (2)	2.08 (2)	2.984 (2)	153 (2)
O1—H2*O*1⋯O2*A* ^i^	0.97 (2)	2.22 (2)	3.105 (2)	150 (2)
O1—H1*O*1⋯O2*B*	0.97 (2)	2.33 (2)	3.297 (10)	170 (2)
O1—H2*O*1⋯O2*B* ^i^	0.97 (2)	2.19 (2)	2.962 (8)	135 (1)
C15—H15⋯*Cg*4^ii^	0.93	2.96	3.730 (2)	141
C27*A*—H27*A*⋯*Cg*2^iii^	0.97	2.86	3.671 (5)	142
C26*B*—H26*D*⋯*Cg*2	0.97	2.89	3.678 (11)	139
C27*B*—H27*D*⋯*Cg*2^iii^	0.97	2.94	3.715 (17)	139

Symmetry codes: (i) 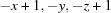; (ii) 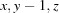; (iii) 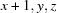.

**Fig. 1.**
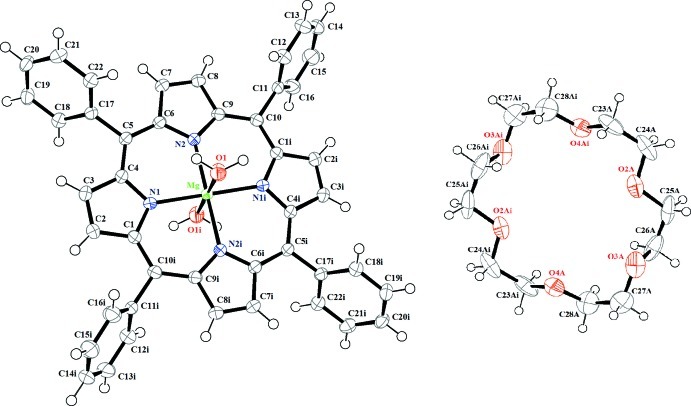
An ORTEP diagram of the structure of [Mg(TPP)(H2O)2].(18-C-6) showing the atom-numbering scheme. Displacement ellipsoids are drawn at 45%. [Symmtry code: (i) -x, -y, -z + 1].

**Fig. 2.**
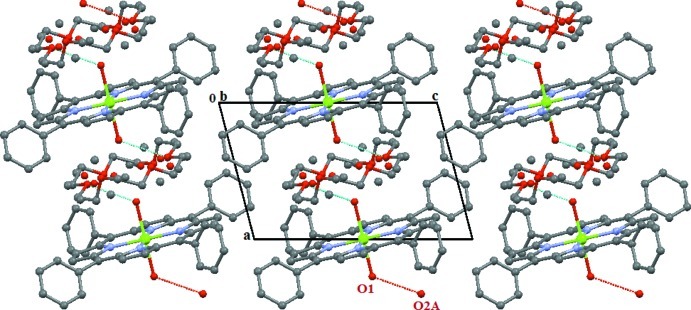
Drawing showing the packing in the lattice of [Mg(TPP)(H2O)2].(18-C-6) viewed down the b axis. H atoms have been omitted for clarity.

## supplementary crystallographic information

### Comment

In continuation of our research on the crystal structures of porphyrin complexes (Belghith *et al.*, 2012) we herein report the synthesis and crystal structure of the bis-aqua-Mg tetraphenylporhyrin derivative [Mg(TPP)(H_2_O)_2_].(18-C-6). In this complex, the coordination geometry of the Mg^2+^ ion is octahedral with four Mg—*N*(pyrrole) bonds in the equatorial porphyrin plane and two Mg—O bonds with the two symmetry related water axial ligands. The average equatorial distance (Mg–Np) equal to 2.071 (1) Å lies in the range [2.065 (4) - 2.092 (7) Å] of the related porphyrin species [Mg(TPP)(4-pic)_2_] (4-pic = 4-picoline: C_6_H_7_N) (McKee *et al.*, 1984) and [Mg(TPP)(H_2_O)] (Choon *et al.*, 1986).

The axial Mg—O(H_2_O) bond length [2.213 (1) Å] is quite longer than in the related derivative [Mg(TPP)(H_2_O)] (2.053 (5) Å) (McKee & Rodley, 1988) but is within the range [2.063 (2) - 2.75 (2) Å] found for several magnesium-aqua non-porphyrin complexes (CSD refcodes DEZNIG; Gryz *et al.*, 2007 and FIVYEP; Imaz *et al.*, 2005) (CDS, version 5.32 Allen, 2002).

The crystal structure of our derivative resembles to one-dimensional coordination polymer where each one of two [Mg(TPP)] moieties is linked to an ether crown 18-C-6 molecule *via* H bonds between the oxygen atom O2A of this species and the O1 atom of the water axial ligand of the [Mg(TPP)(H_2_O)_2_] derivative (Fig. 2).

These linear chains are parallel to the *a* axis and are mainly sustained by weak C—H···*Cg* interactions, where *Cg* is the centroid of the pyrrole or phenyl rings (Table 1).

### Experimental

To a solution of [Mg(TPP)] (15 mg, 0.024 mmol) in chlorobenzene (15 ml) was added an excess of (18-crown-6) (100 mg, 0.378 mmol). The reaction mixture was stirred at room temperature and at the end of the reaction, the color of the solution gradually changes from purple to blue – purple. The resulting material was crystallized by diffusion of hexanes through the chlorobenzene solution which yielded [Mg(TPP)(H_2_O)_2_].(18-C-6). The two water molecules coordinated to the magnesium come from the hygroscopic 18-crown-6 reagent used in excess.

### Refinement

All H atoms were placed in geometrically idealized positions (C—H = 0.93–0.97 Å) and constrained to ride on their parent atoms, with *U*(H) = 1.2*Ueq*(C).

The 18-crown-6 is disordered in two conformations A and B (A is the major conformation) with occupancy coefficients fixed at 80% and 20% respectively.

For the atoms of conformation B, the *DFIX* and SIMU/ISOR restraints (McArdle, 1995) commands in the *SHELXL97* software were used. The *DFIX* constraint instruction was used for some distances in the conformation A: C25A—O2A, C25A—O26A and C23A—C24A while the DANG constraint instruction was also used for the distance C28A—O3A.

### Crystal data

**Table:**

[Mg(C_44_H_28_N_4_)(H_2_O)_2_]·C_12_H_24_O_6_	*Z* = 1
*M**_r_* = 937.36	*F*(000) = 496
Triclinic, *P*1	*D*_x_ = 1.316 Mg m^−^^3^
Hall symbol: -P 1	Mo *K*α radiation, λ = 0.71073 Å
*a* = 8.1440 (3) Å	Cell parameters from 14229 reflections
*b* = 12.3080 (4) Å	θ = 2.9–28.4°
*c* = 12.4170 (4) Å	µ = 0.10 mm^−^^1^
α = 86.894 (3)°	*T* = 180 K
β = 75.163 (3)°	Prism, purple
γ = 79.529 (3)°	0.56 × 0.51 × 0.19 mm
*V* = 1183.06 (7) Å^3^	

### Data collection

**Table:**

Oxford Diffraction Xcalibur (Sapphire1) diffractometer	4650 independent reflections
Radiation source: fine-focus sealed tube	4013 reflections with *I* > 2σ(*I*)
Graphite monochromator	*R*_int_ = 0.031
Detector resolution: 8.2632 pixels mm^-1^	θ_max_ = 26.0°, θ_min_ = 2.9°
ω scans	*h* = −10→10
Absorption correction: multi-scan (*CrysAlis RED*; Oxford Diffraction, 2009)	*k* = −15→15
*T*_min_ = 0.946, *T*_max_ = 0.981	*l* = −15→15
23613 measured reflections	

### Refinement

**Table:**

Refinement on *F*^2^	Primary atom site location: structure-invariant direct methods
Least-squares matrix: full	Secondary atom site location: difference Fourier map
*R*[*F*^2^ > 2σ(*F*^2^)] = 0.044	Hydrogen site location: inferred from neighbouring sites
*wR*(*F*^2^) = 0.118	H atoms treated by a mixture of independent and constrained refinement
*S* = 1.04	*w* = 1/[σ^2^(*F*_o_^2^) + (0.0568*P*)^2^ + 0.6503*P*] where *P* = (*F*_o_^2^ + 2*F*_c_^2^)/3
4650 reflections	(Δ/σ)_max_ < 0.001
400 parameters	Δρ_max_ = 0.46 e Å^−^^3^
119 restraints	Δρ_min_ = −0.28 e Å^−^^3^

### Special details

**Table:**

Geometry. All s.u.'s (except the s.u. in the dihedral angle between two l.s. planes) are estimated using the full covariance matrix. The cell s.u.'s are taken into account individually in the estimation of s.u.'s in distances, angles and torsion angles; correlations between s.u.'s in cell parameters are only used when they are defined by crystal symmetry. An approximate (isotropic) treatment of cell s.u.'s is used for estimating s.u.'s involving l.s. planes.
Refinement. Refinement of *F*^2^ against ALL reflections. The weighted *R*-factor *wR* and goodness of fit *S* are based on *F*^2^, conventional *R*-factors *R* are based on *F*, with *F* set to zero for negative *F*^2^. The threshold expression of *F*^2^ > σ(*F*^2^) is used only for calculating *R*-factors(gt) *etc*. and is not relevant to the choice of reflections for refinement. *R*-factors based on *F*^2^ are statistically about twice as large as those based on *F*, and *R*- factors based on ALL data will be even larger.

### Fractional atomic coordinates and isotropic or equivalent isotropic displacement parameters (Å^2^)

**Table:**

	*x*	*y*	*z*	*U*_iso_*/*U*_eq_	Occ. (<1)
Mg1	0.0000	0.0000	0.5000	0.01893 (17)	
O1	0.27384 (14)	−0.00267 (10)	0.49625 (10)	0.0273 (3)	
H1O1	0.323 (3)	−0.0256 (18)	0.5592 (13)	0.050*	
H2O1	0.360 (2)	−0.0260 (18)	0.4278 (12)	0.050*	
N1	−0.05837 (16)	−0.03608 (10)	0.66901 (10)	0.0189 (3)	
N2	−0.06769 (16)	0.16696 (10)	0.53555 (10)	0.0185 (3)	
C1	−0.04664 (19)	−0.13929 (12)	0.71606 (12)	0.0197 (3)	
C2	−0.1086 (2)	−0.12941 (13)	0.83554 (13)	0.0253 (3)	
H2	−0.1120	−0.1871	0.8872	0.030*	
C3	−0.1607 (2)	−0.02077 (13)	0.85834 (13)	0.0256 (3)	
H3	−0.2093	0.0103	0.9284	0.031*	
C4	−0.12687 (19)	0.03831 (12)	0.75337 (12)	0.0203 (3)	
C5	−0.15851 (19)	0.15423 (12)	0.74036 (12)	0.0202 (3)	
C6	−0.12549 (19)	0.21291 (12)	0.63867 (12)	0.0198 (3)	
C7	−0.1471 (2)	0.33201 (13)	0.62737 (13)	0.0239 (3)	
H7	−0.1822	0.3827	0.6851	0.029*	
C8	−0.1069 (2)	0.35562 (12)	0.51726 (13)	0.0234 (3)	
H8	−0.1091	0.4256	0.4848	0.028*	
C9	−0.05984 (19)	0.25119 (12)	0.45940 (12)	0.0193 (3)	
C10	0.01238 (19)	−0.23950 (12)	0.65711 (12)	0.0198 (3)	
C11	0.0337 (2)	−0.34328 (12)	0.72424 (12)	0.0215 (3)	
C12	0.1988 (2)	−0.40022 (13)	0.71999 (14)	0.0277 (4)	
H12	0.2936	−0.3734	0.6755	0.033*	
C13	0.2240 (3)	−0.49656 (14)	0.78129 (15)	0.0346 (4)	
H13	0.3353	−0.5339	0.7777	0.042*	
C14	0.0846 (3)	−0.53712 (14)	0.84755 (15)	0.0355 (4)	
H14	0.1015	−0.6014	0.8892	0.043*	
C15	−0.0798 (3)	−0.48205 (15)	0.85186 (15)	0.0373 (4)	
H15	−0.1743	−0.5097	0.8958	0.045*	
C16	−0.1050 (2)	−0.38552 (14)	0.79099 (14)	0.0311 (4)	
H16	−0.2166	−0.3485	0.7950	0.037*	
C17	−0.2409 (2)	0.22080 (12)	0.84322 (12)	0.0214 (3)	
C18	−0.1582 (2)	0.22428 (13)	0.92774 (13)	0.0262 (3)	
H18	−0.0482	0.1834	0.9210	0.031*	
C19	−0.2371 (2)	0.28757 (14)	1.02156 (13)	0.0295 (4)	
H19	−0.1797	0.2894	1.0771	0.035*	
C20	−0.4005 (2)	0.34789 (14)	1.03309 (13)	0.0296 (4)	
H20	−0.4535	0.3906	1.0961	0.036*	
C21	−0.4850 (2)	0.34448 (14)	0.95063 (14)	0.0319 (4)	
H21	−0.5958	0.3844	0.9585	0.038*	
C22	−0.4054 (2)	0.28192 (14)	0.85624 (14)	0.0275 (4)	
H22	−0.4631	0.2808	0.8007	0.033*	
C23A	0.5253 (4)	−0.1823 (3)	0.7059 (2)	0.0620 (12)	0.80
H23A	0.6378	−0.1605	0.6826	0.074*	0.80
H23B	0.5341	−0.2468	0.7539	0.074*	0.80
O4A	0.4741 (2)	−0.20837 (18)	0.61214 (18)	0.0495 (5)	0.80
C24A	0.3972 (4)	−0.0906 (3)	0.7682 (2)	0.0682 (10)	0.80
H24A	0.2828	−0.1097	0.7866	0.082*	0.80
H24B	0.4258	−0.0775	0.8370	0.082*	0.80
O2A	0.4010 (3)	0.0049 (2)	0.70026 (15)	0.0583 (5)	0.80
C25A	0.3047 (6)	0.1004 (4)	0.7585 (5)	0.0708 (16)	0.80
H25A	0.3639	0.1206	0.8110	0.085*	0.80
H25B	0.1925	0.0859	0.8001	0.085*	0.80
C26A	0.2830 (3)	0.1925 (3)	0.6784 (3)	0.0665 (10)	0.80
H26A	0.2331	0.1705	0.6218	0.080*	0.80
H26B	0.2067	0.2563	0.7168	0.080*	0.80
C27A	0.4410 (6)	0.3090 (3)	0.5543 (3)	0.0657 (11)	0.80
H27A	0.5529	0.3318	0.5349	0.079*	0.80
H27B	0.3573	0.3703	0.5919	0.079*	0.80
C28A	0.3963 (4)	0.2866 (3)	0.4501 (2)	0.0658 (9)	0.80
H28A	0.2878	0.2595	0.4680	0.079*	0.80
H28B	0.3831	0.3543	0.4071	0.079*	0.80
O3A	0.4463 (2)	0.2189 (2)	0.62918 (17)	0.0605 (6)	0.80
C23B	0.485 (2)	−0.1672 (8)	0.7359 (12)	0.0502 (12)	0.20
H23C	0.3729	−0.1875	0.7676	0.060*	0.20
H23D	0.5497	−0.1783	0.7925	0.060*	0.20
C24B	0.3936 (16)	0.0228 (9)	0.7901 (8)	0.0497 (11)	0.20
H24C	0.4858	0.0447	0.8157	0.060*	0.20
H24D	0.3206	−0.0108	0.8523	0.060*	0.20
C25B	0.290 (2)	0.1223 (19)	0.750 (2)	0.0485 (11)	0.20
H25C	0.2036	0.1007	0.7179	0.058*	0.20
H25D	0.2324	0.1728	0.8103	0.058*	0.20
C26B	0.3267 (13)	0.2581 (8)	0.6048 (9)	0.0466 (11)	0.20
H26C	0.2533	0.3143	0.6558	0.056*	0.20
H26D	0.2538	0.2265	0.5689	0.056*	0.20
C27B	0.453 (2)	0.3115 (11)	0.5178 (11)	0.0464 (12)	0.20
H27C	0.3923	0.3764	0.4874	0.056*	0.20
H27D	0.5364	0.3346	0.5508	0.056*	0.20
C28B	0.5792 (17)	−0.2380 (10)	0.6372 (11)	0.0500 (13)	0.20
H28C	0.6817	−0.2096	0.5971	0.060*	0.20
H28D	0.6139	−0.3131	0.6611	0.060*	0.20
O2B	0.4649 (11)	−0.0550 (7)	0.7021 (7)	0.0507 (11)	0.20
O3B	0.4086 (12)	0.1731 (7)	0.6670 (7)	0.0473 (10)	0.20
O4B	0.5366 (12)	0.2356 (7)	0.4328 (7)	0.0463 (12)	0.20

### Atomic displacement parameters (Å^2^)

**Table:**

	*U*^11^	*U*^22^	*U*^33^	*U*^12^	*U*^13^	*U*^23^
Mg1	0.0259 (4)	0.0142 (3)	0.0152 (3)	−0.0009 (3)	−0.0041 (3)	−0.0010 (3)
O1	0.0240 (6)	0.0324 (6)	0.0247 (6)	−0.0030 (5)	−0.0060 (5)	−0.0011 (5)
N1	0.0217 (6)	0.0157 (6)	0.0181 (6)	0.0000 (5)	−0.0050 (5)	−0.0011 (5)
N2	0.0218 (6)	0.0166 (6)	0.0162 (6)	−0.0011 (5)	−0.0047 (5)	−0.0009 (5)
C1	0.0203 (7)	0.0195 (7)	0.0189 (7)	−0.0011 (6)	−0.0060 (6)	0.0010 (6)
C2	0.0327 (9)	0.0228 (8)	0.0183 (8)	−0.0021 (6)	−0.0050 (6)	0.0026 (6)
C3	0.0340 (9)	0.0235 (8)	0.0163 (7)	−0.0016 (7)	−0.0030 (6)	−0.0012 (6)
C4	0.0222 (7)	0.0205 (7)	0.0171 (7)	−0.0005 (6)	−0.0045 (6)	−0.0022 (6)
C5	0.0221 (7)	0.0193 (7)	0.0185 (7)	−0.0002 (6)	−0.0057 (6)	−0.0034 (6)
C6	0.0212 (7)	0.0184 (7)	0.0195 (7)	−0.0007 (6)	−0.0061 (6)	−0.0030 (6)
C7	0.0309 (8)	0.0180 (7)	0.0224 (8)	−0.0007 (6)	−0.0075 (6)	−0.0043 (6)
C8	0.0315 (8)	0.0154 (7)	0.0237 (8)	−0.0029 (6)	−0.0084 (6)	−0.0010 (6)
C9	0.0202 (7)	0.0169 (7)	0.0206 (7)	−0.0015 (5)	−0.0061 (6)	−0.0003 (6)
C10	0.0201 (7)	0.0182 (7)	0.0208 (7)	−0.0020 (6)	−0.0058 (6)	0.0015 (6)
C11	0.0315 (8)	0.0163 (7)	0.0170 (7)	−0.0025 (6)	−0.0074 (6)	−0.0017 (6)
C12	0.0316 (9)	0.0249 (8)	0.0265 (8)	−0.0020 (7)	−0.0092 (7)	0.0017 (7)
C13	0.0434 (10)	0.0252 (9)	0.0355 (10)	0.0053 (7)	−0.0183 (8)	0.0001 (7)
C14	0.0617 (12)	0.0185 (8)	0.0255 (9)	−0.0017 (8)	−0.0139 (8)	0.0036 (7)
C15	0.0500 (11)	0.0271 (9)	0.0297 (9)	−0.0097 (8)	−0.0001 (8)	0.0056 (7)
C16	0.0330 (9)	0.0263 (8)	0.0300 (9)	−0.0026 (7)	−0.0030 (7)	0.0038 (7)
C17	0.0282 (8)	0.0164 (7)	0.0178 (7)	−0.0024 (6)	−0.0034 (6)	−0.0006 (6)
C18	0.0286 (8)	0.0265 (8)	0.0220 (8)	0.0001 (6)	−0.0067 (6)	−0.0022 (6)
C19	0.0400 (10)	0.0301 (9)	0.0192 (8)	−0.0059 (7)	−0.0085 (7)	−0.0024 (6)
C20	0.0414 (10)	0.0233 (8)	0.0187 (8)	−0.0019 (7)	0.0007 (7)	−0.0047 (6)
C21	0.0314 (9)	0.0286 (9)	0.0290 (9)	0.0063 (7)	−0.0027 (7)	−0.0054 (7)
C22	0.0308 (9)	0.0269 (8)	0.0235 (8)	0.0010 (7)	−0.0083 (7)	−0.0039 (6)
C23A	0.042 (2)	0.091 (3)	0.054 (3)	−0.0160 (18)	−0.0204 (18)	0.040 (2)
O4A	0.0354 (10)	0.0570 (13)	0.0543 (13)	−0.0082 (9)	−0.0101 (9)	0.0096 (10)
C24A	0.060 (2)	0.115 (3)	0.0319 (14)	−0.032 (2)	−0.0086 (13)	0.0206 (17)
O2A	0.0538 (13)	0.0805 (16)	0.0346 (10)	−0.0104 (11)	−0.0002 (9)	−0.0045 (10)
C25A	0.047 (2)	0.106 (5)	0.053 (3)	−0.029 (3)	0.0190 (19)	−0.045 (3)
C26A	0.0301 (15)	0.077 (2)	0.089 (3)	−0.0051 (14)	−0.0012 (15)	−0.050 (2)
C27A	0.056 (2)	0.068 (2)	0.078 (3)	−0.0151 (16)	−0.020 (2)	−0.008 (2)
C28A	0.0435 (17)	0.076 (2)	0.076 (2)	−0.0041 (15)	−0.0145 (16)	−0.0034 (18)
O3A	0.0388 (11)	0.0919 (18)	0.0554 (13)	−0.0145 (11)	−0.0143 (10)	−0.0184 (13)
C23B	0.0502 (13)	0.0501 (13)	0.0497 (13)	−0.0081 (7)	−0.0120 (7)	0.0002 (7)
C24B	0.0495 (12)	0.0499 (12)	0.0491 (12)	−0.0080 (7)	−0.0117 (7)	0.0003 (7)
C25B	0.0482 (12)	0.0488 (12)	0.0483 (12)	−0.0084 (7)	−0.0120 (7)	−0.0004 (7)
C26B	0.0461 (12)	0.0469 (12)	0.0472 (12)	−0.0086 (7)	−0.0120 (7)	−0.0007 (7)
C27B	0.0459 (13)	0.0468 (13)	0.0471 (13)	−0.0086 (7)	−0.0125 (7)	0.0001 (7)
C28B	0.0501 (14)	0.0499 (14)	0.0498 (14)	−0.0082 (8)	−0.0125 (8)	−0.0002 (8)
O2B	0.0504 (12)	0.0504 (12)	0.0499 (12)	−0.0074 (7)	−0.0112 (7)	0.0010 (7)
O3B	0.0470 (12)	0.0478 (12)	0.0474 (12)	−0.0088 (7)	−0.0117 (7)	−0.0008 (7)
O4B	0.0457 (14)	0.0465 (14)	0.0471 (14)	−0.0084 (8)	−0.0127 (8)	0.0009 (8)

### Geometric parameters (Å, º)

**Table:**

Mg1—N2	2.0697 (12)	C21—C22	1.383 (2)
Mg1—N2^i^	2.0697 (12)	C21—H21	0.9300
Mg1—N1	2.0717 (12)	C22—H22	0.9300
Mg1—N1^i^	2.0717 (12)	C23A—O4A	1.404 (3)
Mg1—O1	2.2130 (11)	C23A—C24A	1.488 (4)
Mg1—O1^i^	2.2130 (11)	C23A—H23A	0.9700
O1—H1O1	0.972 (10)	C23A—H23B	0.9700
O1—H2O1	0.972 (10)	O4A—C28A^ii^	1.389 (4)
N1—C4	1.3659 (19)	C24A—O2A	1.410 (3)
N1—C1	1.3669 (18)	C24A—H24A	0.9700
N2—C6	1.3615 (19)	C24A—H24B	0.9700
N2—C9	1.3638 (19)	O2A—C25A	1.410 (5)
C1—C10	1.411 (2)	C25A—C26A	1.484 (3)
C1—C2	1.444 (2)	C25A—H25A	0.9700
C2—C3	1.350 (2)	C25A—H25B	0.9700
C2—H2	0.9300	C26A—O3A	1.402 (3)
C3—C4	1.446 (2)	C26A—H26A	0.9700
C3—H3	0.9300	C26A—H26B	0.9700
C4—C5	1.411 (2)	C27A—O3A	1.409 (3)
C5—C6	1.409 (2)	C27A—C28A	1.483 (3)
C5—C17	1.493 (2)	C27A—H27A	0.9700
C6—C7	1.448 (2)	C27A—H27B	0.9700
C7—C8	1.350 (2)	C28A—O4A^ii^	1.389 (4)
C7—H7	0.9300	C28A—H28A	0.9700
C8—C9	1.447 (2)	C28A—H28B	0.9700
C8—H8	0.9300	C23B—O2B	1.413 (6)
C9—C10^i^	1.407 (2)	C23B—C28B	1.497 (6)
C10—C9^i^	1.407 (2)	C23B—H23C	0.9700
C10—C11	1.495 (2)	C23B—H23D	0.9700
C11—C16	1.386 (2)	C24B—O2B	1.427 (6)
C11—C12	1.388 (2)	C24B—C25B	1.497 (6)
C12—C13	1.386 (2)	C24B—H24C	0.9700
C12—H12	0.9300	C24B—H24D	0.9700
C13—C14	1.378 (3)	C25B—O3B	1.420 (6)
C13—H13	0.9300	C25B—H25C	0.9700
C14—C15	1.375 (3)	C25B—H25D	0.9700
C14—H14	0.9300	C26B—O3B	1.435 (6)
C15—C16	1.385 (2)	C26B—C27B	1.500 (6)
C15—H15	0.9300	C26B—H26C	0.9700
C16—H16	0.9300	C26B—H26D	0.9700
C17—C22	1.387 (2)	C27B—O4B	1.400 (6)
C17—C18	1.391 (2)	C27B—H27C	0.9700
C18—C19	1.382 (2)	C27B—H27D	0.9700
C18—H18	0.9300	C28B—O4B^ii^	1.433 (15)
C19—C20	1.377 (2)	C28B—H28C	0.9700
C19—H19	0.9300	C28B—H28D	0.9700
C20—C21	1.378 (3)	O4B—C28B^ii^	1.433 (15)
C20—H20	0.9300		
			
N2—Mg1—N2^i^	180.0	C20—C21—C22	120.24 (16)
N2—Mg1—N1	89.79 (5)	C20—C21—H21	119.9
N2^i^—Mg1—N1	90.21 (5)	C22—C21—H21	119.9
N2—Mg1—N1^i^	90.21 (5)	C21—C22—C17	120.94 (15)
N2^i^—Mg1—N1^i^	89.79 (5)	C21—C22—H22	119.5
N1—Mg1—N1^i^	180.0	C17—C22—H22	119.5
N2—Mg1—O1	92.86 (5)	O4A—C23A—C24A	109.8 (3)
N2^i^—Mg1—O1	87.14 (5)	O4A—C23A—H23A	109.7
N1—Mg1—O1	91.48 (4)	C24A—C23A—H23A	109.7
N1^i^—Mg1—O1	88.52 (4)	O4A—C23A—H23B	109.7
N2—Mg1—O1^i^	87.14 (5)	C24A—C23A—H23B	109.7
N2^i^—Mg1—O1^i^	92.86 (5)	H23A—C23A—H23B	108.2
N1—Mg1—O1^i^	88.52 (4)	C28A^ii^—O4A—C23A	108.6 (2)
N1^i^—Mg1—O1^i^	91.48 (4)	O2A—C24A—C23A	108.2 (2)
O1—Mg1—O1^i^	180.0	O2A—C24A—H24A	110.1
Mg1—O1—H1O1	123.5 (14)	C23A—C24A—H24A	110.1
Mg1—O1—H2O1	117.8 (14)	O2A—C24A—H24B	110.1
H1O1—O1—H2O1	108.9 (19)	C23A—C24A—H24B	110.1
C4—N1—C1	107.47 (12)	H24A—C24A—H24B	108.4
C4—N1—Mg1	126.28 (10)	C24A—O2A—C25A	112.3 (3)
C1—N1—Mg1	126.10 (10)	O2A—C25A—C26A	109.6 (4)
C6—N2—C9	107.51 (12)	O2A—C25A—H25A	109.8
C6—N2—Mg1	126.51 (10)	C26A—C25A—H25A	109.8
C9—N2—Mg1	125.97 (10)	O2A—C25A—H25B	109.8
N1—C1—C10	125.43 (13)	C26A—C25A—H25B	109.8
N1—C1—C2	109.09 (13)	H25A—C25A—H25B	108.2
C10—C1—C2	125.46 (14)	O3A—C26A—C25A	107.9 (3)
C3—C2—C1	107.21 (13)	O3A—C26A—H26A	110.1
C3—C2—H2	126.4	C25A—C26A—H26A	110.1
C1—C2—H2	126.4	O3A—C26A—H26B	110.1
C2—C3—C4	107.15 (14)	C25A—C26A—H26B	110.1
C2—C3—H3	126.4	H26A—C26A—H26B	108.4
C4—C3—H3	126.4	O3A—C27A—C28A	114.9 (3)
N1—C4—C5	125.54 (13)	O3A—C27A—H27A	108.5
N1—C4—C3	109.05 (13)	C28A—C27A—H27A	108.5
C5—C4—C3	125.41 (14)	O3A—C27A—H27B	108.5
C6—C5—C4	126.02 (14)	C28A—C27A—H27B	108.5
C6—C5—C17	116.87 (13)	H27A—C27A—H27B	107.5
C4—C5—C17	117.07 (13)	O4A^ii^—C28A—C27A	109.7 (3)
N2—C6—C5	125.61 (13)	O4A^ii^—C28A—H28A	109.7
N2—C6—C7	109.11 (13)	C27A—C28A—H28A	109.7
C5—C6—C7	125.28 (14)	O4A^ii^—C28A—H28B	109.7
C8—C7—C6	107.17 (14)	C27A—C28A—H28B	109.7
C8—C7—H7	126.4	H28A—C28A—H28B	108.2
C6—C7—H7	126.4	C26A—O3A—C27A	113.0 (3)
C7—C8—C9	106.88 (13)	O2B—C23B—C28B	109.3 (10)
C7—C8—H8	126.6	O2B—C23B—H23C	109.8
C9—C8—H8	126.6	C28B—C23B—H23C	109.8
N2—C9—C10^i^	125.84 (13)	O2B—C23B—H23D	109.8
N2—C9—C8	109.24 (13)	C28B—C23B—H23D	109.8
C10^i^—C9—C8	124.92 (14)	H23C—C23B—H23D	108.3
C9^i^—C10—C1	126.30 (14)	O2B—C24B—C25B	109.7 (16)
C9^i^—C10—C11	116.47 (13)	O2B—C24B—H24C	109.7
C1—C10—C11	117.18 (13)	C25B—C24B—H24C	109.7
C16—C11—C12	118.25 (14)	O2B—C24B—H24D	109.7
C16—C11—C10	122.71 (14)	C25B—C24B—H24D	109.7
C12—C11—C10	119.04 (14)	H24C—C24B—H24D	108.2
C13—C12—C11	120.79 (16)	O3B—C25B—C24B	106.3 (10)
C13—C12—H12	119.6	O3B—C25B—H25C	110.5
C11—C12—H12	119.6	C24B—C25B—H25C	110.5
C14—C13—C12	120.16 (17)	O3B—C25B—H25D	110.5
C14—C13—H13	119.9	C24B—C25B—H25D	110.5
C12—C13—H13	119.9	H25C—C25B—H25D	108.7
C15—C14—C13	119.68 (16)	O3B—C26B—C27B	113.2 (12)
C15—C14—H14	120.2	O3B—C26B—H26C	108.9
C13—C14—H14	120.2	C27B—C26B—H26C	108.9
C14—C15—C16	120.15 (17)	O3B—C26B—H26D	108.9
C14—C15—H15	119.9	C27B—C26B—H26D	108.9
C16—C15—H15	119.9	H26C—C26B—H26D	107.8
C15—C16—C11	120.96 (17)	O4B—C27B—C26B	109.0 (10)
C15—C16—H16	119.5	O4B—C27B—H27C	109.9
C11—C16—H16	119.5	C26B—C27B—H27C	109.9
C22—C17—C18	118.08 (14)	O4B—C27B—H27D	109.9
C22—C17—C5	119.67 (14)	C26B—C27B—H27D	109.9
C18—C17—C5	122.25 (14)	H27C—C27B—H27D	108.3
C19—C18—C17	120.96 (15)	O4B^ii^—C28B—C23B	107.2 (13)
C19—C18—H18	119.5	O4B^ii^—C28B—H28C	110.3
C17—C18—H18	119.5	C23B—C28B—H28C	110.3
C20—C19—C18	120.19 (16)	O4B^ii^—C28B—H28D	110.3
C20—C19—H19	119.9	C23B—C28B—H28D	110.3
C18—C19—H19	119.9	H28C—C28B—H28D	108.5
C19—C20—C21	119.59 (15)	C23B—O2B—C24B	115.3 (10)
C19—C20—H20	120.2	C25B—O3B—C26B	113.4 (10)
C21—C20—H20	120.2	C27B—O4B—C28B^ii^	106.0 (12)

Symmetry codes: (i) −*x*, −*y*, −*z*+1; (ii) −*x*+1, −*y*, −*z*+1.

### Hydrogen-bond geometry (Å, º)

Cg2 and Cg4 are the centroids of the N2/C6–C9 and C17–C22 rings, respectively.

**Table:**

*D*—H···*A*	*D*—H	H···*A*	*D*···*A*	*D*—H···*A*
O1—H1*O*1···O2*A*	0.97 (2)	2.08 (2)	2.984 (2)	153 (2)
O1—H2*O*1···O2*A*^ii^	0.97 (2)	2.22 (2)	3.105 (2)	150 (2)
O1—H1*O*1···O2*B*	0.97 (2)	2.33 (2)	3.297 (10)	170 (2)
O1—H2*O*1···O2*B*^ii^	0.97 (2)	2.19 (2)	2.962 (8)	135 (1)
C15—H15···*Cg*4^iii^	0.93	2.96	3.730 (2)	141
C27*A*—H27*A*···*Cg*2^iv^	0.97	2.86	3.671 (5)	142
C26*B*—H26*D*···*Cg*2	0.97	2.89	3.678 (11)	139
C27*B*—H27*D*···*Cg*2^iv^	0.97	2.94	3.715 (17)	139

Symmetry codes: (ii) −*x*+1, −*y*, −*z*+1; (iii) *x*, *y*−1, *z*; (iv) *x*+1, *y*, *z*.
